# Research on Estimating Rice Canopy Height and LAI Based on LiDAR Data

**DOI:** 10.3390/s23198334

**Published:** 2023-10-09

**Authors:** Linlong Jing, Xinhua Wei, Qi Song, Fei Wang

**Affiliations:** Key Laboratory of Modern Agricultural Equipment and Technology, Ministry of Education of the People’s Republic of China, Institute of Agricultural Engineering, Jiangsu University, Zhenjiang 212013, China

**Keywords:** rice canopy height and density, LiDAR, rice canopy LAI, regression analysis

## Abstract

Rice canopy height and density are directly usable crop phenotypic traits for the direct estimation of crop biomass. Therefore, it is crucial to rapidly and accurately estimate these phenotypic parameters. To achieve the non-destructive detection and estimation of these essential parameters in rice, a platform based on LiDAR (Light Detection and Ranging) point cloud data for rice phenotypic parameter detection was established. Data collection of rice canopy layers was performed across multiple plots. The LiDAR-detected canopy-top point clouds were selected using a method based on the highest percentile, and a surface model of the canopy was calculated. The canopy height estimation was the difference between the ground elevation and the percentile value. To determine the optimal percentile that would define the rice canopy top, testing was conducted incrementally at percentile values from 0.8 to 1, with increments of 0.005. The optimal percentile value was found to be 0.975. The root mean square error (RMSE) between the LiDAR-detected and manually measured canopy heights for each case was calculated. The prediction model based on canopy height (R^2^ = 0.941, RMSE = 0.019) exhibited a strong correlation with the actual canopy height. Linear regression analysis was conducted between the gap fractions of different plots, and the average rice canopy Leaf Area Index (LAI) was manually detected. Prediction models of canopy LAIs based on ground return counts (R^2^ = 0.24, RMSE = 0.1) and ground return intensity (R^2^ = 0.28, RMSE = 0.09) showed strong correlations but had lower correlations with rice canopy LAIs. Regression analysis was performed between LiDAR-detected canopy heights and manually measured rice canopy LAIs. The results thereof indicated that the prediction model based on canopy height (R^2^ = 0.77, RMSE = 0.03) was more accurate.

## 1. Introduction

Rice is one of the world’s major staple crops, as it constitutes the primary diet for over half of the global population [1,2]. Crop protection operations in rice cultivation are a crucial aspect of rice management due to the occurrence of pests and diseases throughout the plant’s lifecycle. During pesticide application, rice canopy parameters can significantly influence the dispersion and retention of droplets [3,4]. The deposition of spray droplets in dense canopies has been 40% lower than in canopies with moderate and low densities [5]. Consequently, the effective detection of rice canopy density can offer valuable insights for the calculation of spray quantities during variable-rate application processes.

The primary metrics for assessing rice canopy density include the LAI [6] and plant height. The LAI represents the total area of plant leaves per unit of ground surface area [7,8]. A higher LAI value indicates a canopy with a greater leaf area density, signifying a higher canopy density. Field surveys have revealed that rice canopy density undergoes significant changes from the tillering to the jointing stage, with LAIs increasing rapidly during this period. After jointing, the trend of LAI variation will become more stable and canopy density changes will be less pronounced [9].

Plant height is a key trait that affects the yield potential of rice [10]. It refers to the vertical distance from the ground to the top of the plant. Crop height can directly reflect the growth status of the crop and be used to estimate its biomass [11]. Traditional destructive sampling to assess LAI is both labor-intensive and time-consuming, making it unsuitable for measuring a large number of crop samples. Various non-destructive methods for estimating LAI have inevitably introduced measurement errors associated with operators. Currently, there is no established method for non-destructively estimating crop LAIs throughout the entire growing season [12], and a similar issue exists for the measurement of plant height. However, there is a significant difference in the relationship between LAI and vegetation height for various vegetation types and heights [13,14].

Various sensing technologies, such as RGB cameras [15], ultrasonic sensors [16], multispectral and hyperspectral sensors [17] and laser scanners [18], have been utilized to characterize crop canopies [19]. Stereo vision technology has been employed to describe intricate geometric features and extend traditional two-dimensional (2D) imaging methods in order to characterize three-dimensional (3D) plant structures using multiview images [20]. However, these techniques have certain drawbacks, including stereo matching errors caused by lighting and shadows, incomplete reconstructed data due to occlusion between plant components and a trade-off between accuracy and efficiency [21].

Ultrasonic sensors are relatively inexpensive and easy to use, but they often have lower measurement accuracy and are susceptible to interference from their surroundings and issues based on measurement distance. Spectral images provide rich spectral and texture information but lack structural information that would aid in understanding plant functionality. Optical images can effectively extract specific 3D phenotypic features (such as leaf area, canopy size and plant height) in controlled environments. However, accurately and comprehensively characterizing 3D plant structures in outdoor environments often presents limitations.

The emergence of optical detection and LiDAR technology has provided a powerful tool for the acquisition of 3D structural crop data. LiDAR technology actively emits laser pulses and measures the distance to each target using time-of-flight principles. It has found wide applications in forest resource surveys [22] and ecosystem monitoring [23]. The laser beam emitted by a LiDAR sensor has advantages such as high energy density, a small divergence angle and a long linear propagation distance. It can partially penetrate vegetation canopies and overcome challenges associated with image-based phenotyping, such as illumination and saturation effects. Recently, LiDAR has gained increased attention in plant phenotyping research [24]. LiDAR systems installed on unmanned aerial vehicles or ground platforms can provide accurate measurements of the phenotypic traits of crops such as peanuts, maize and fruit trees at the regional level [25,26,27].

However, these crops are more inclined to grow in the early stages, with clear row spacing and less leaf overlap, thus possessing distinct crop features. In contrast, the rice canopy will become dense in the mid to late growth stages, making it difficult to distinguish planting rows. Rice is cultivated in paddies, making it challenging for conventional ground platforms to access the fields. Moreover, water-filled channels and depressions in the paddy fields make it difficult for ground platforms and LiDAR systems to maintain smooth movement. The airflow generated by drones can disturb the rice canopy. These factors collectively increase the difficulty of using LiDAR technology to measure the dense canopy heights and LAIs of rice in the later growth stages. Therefore, the primary objective of this research was to utilize LiDAR technology in paddy fields to estimate these parameters, with the aim to provide support for the sustainable development of the variable-rate spraying and agricultural production of rice.

## 2. Materials and Methods

### 2.1. Plant Materials

**Figure 1:**
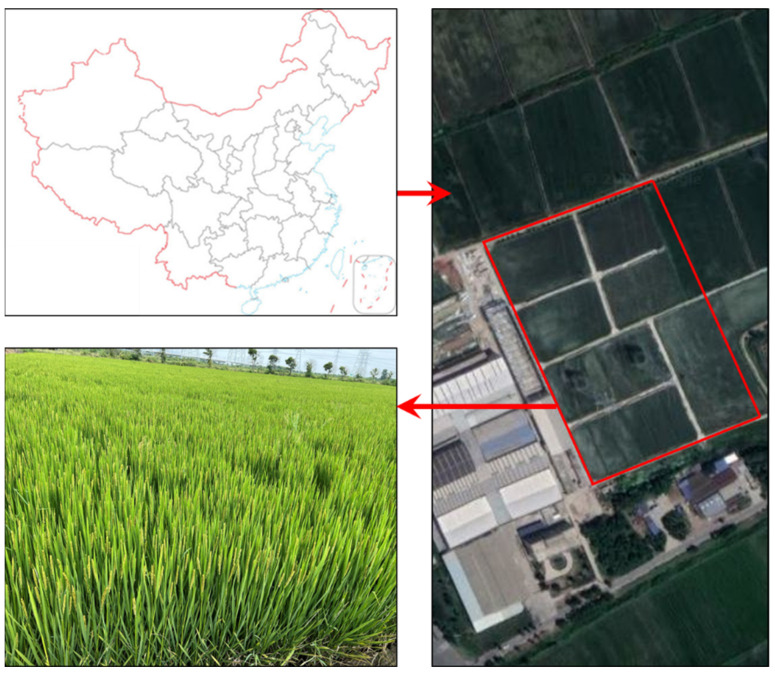
Rice sampling field.

This experiment was conducted in a rice field located in the Runguo Agricultural Base in Zhenjiang City, Jiangsu Province, China, in the year 2022. The geographical coordinates of this area are approximately 32°54′19″ N in latitude and 116°23′28″ E in longitude (Figure 1). The rice sample variety used in this study was “Nanjing 9108”. This rice was in the heading stage of growth during this experiment, with a planting configuration of 18 cm between rows and 10 cm between individual plants within a row.

### 2.2. System Architecture

**Figure 2:**
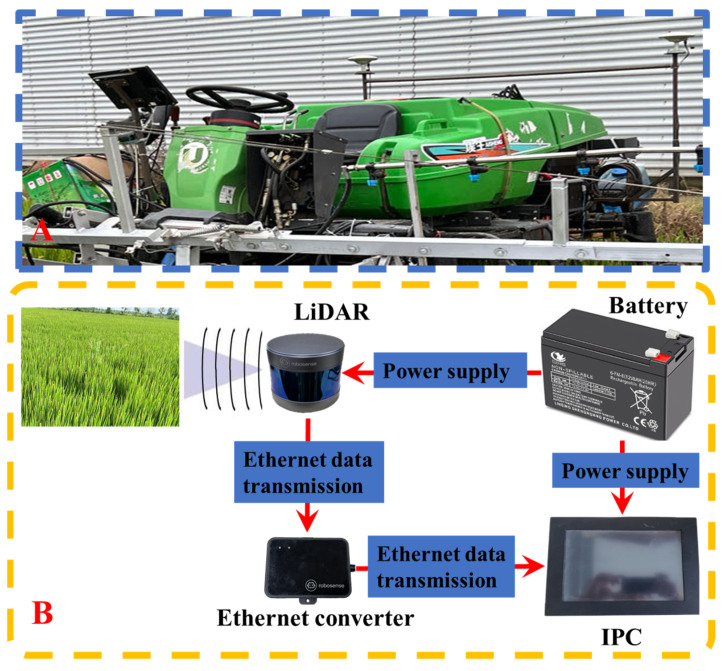
Point cloud data acquisition platform and system. (**A**) Point cloud data acquisition platform. (**B**) Point cloud data acquisition system.

The LiDAR data collection platform (Figure 2A) consisted of a spray boom sprayer (Swan Group Essen SWAN3WP-500) and a framework equipped with a ground-based LiDAR sensor (RS-LiDAR-16; SICK AG, Waldkirch, Germany). This setup also included an industrial control unit, a battery unit (24 V, 18.0 Amp.Hr.) and other necessary cables. The specifications of the LiDAR are presented in Table 1. The specifications of the spray boom sprayer are presented in Table 2.

The LiDAR was mounted on a high-clearance sprayer platform situated 1.5 m above the ground (Figure 2A). Due to the front installation of the boom of the spraying machine, to obtain a larger scanning area and avoid the obstruction of the spray boom and other devices to the LiDAR, the installation angle of the LiDAR was determined to be horizontal and inclined downward by 30°. To obtain a comprehensive view of the entire rice canopy, the LiDAR was configured to operate in a continuous line-scanning mode, covering a 360° field of view with a resolution of 0.09°. For each line scan, the LiDAR output 320,000 points per second. To ensure high-speed data collection, the LiDAR was connected to an industrial control unit (Figure 2B) via a serial-to-Ethernet converter. The LiDAR scan data, including distance, angle and reflectivity information, were encapsulated into packets using the Main Stream Output Protocol (MSOP) and sent to the industrial control unit. RSVIEW 1.4.3 was employed for point cloud data collection and storage as well as communication with the LiDAR. It correctly received data packets, extracted point cloud data, converted polar coordinates into Cartesian coordinates and eventually saved the data in a Pcap file. All line scan data were timestamped with distance and time information.

### 2.3. Field Setup and Data Acquisition

**Figure 3:**
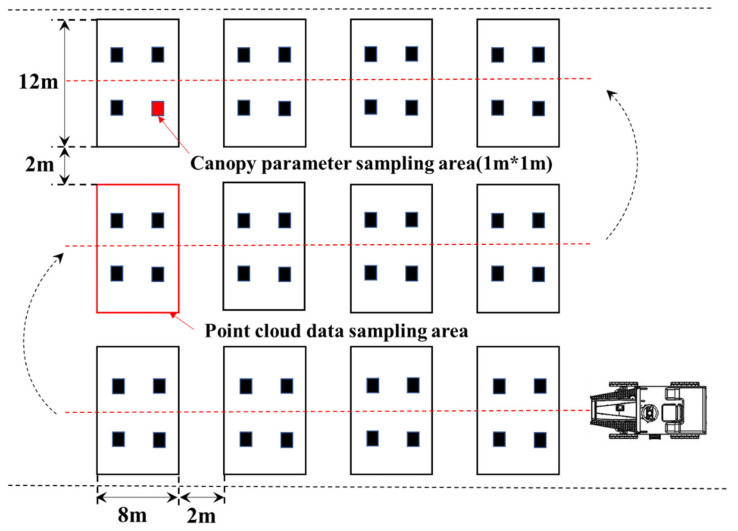
The distribution of rice canopy point cloud data collection plots.

The point cloud data collection platform was utilized to gather information about the rice canopy in the designated field. The sprayer machine traveled along the planting rows, minimizing damage to the rice plants and ensuring that the LiDAR’s detection of rice canopy information remained unaffected. Each field plot was 12 m wide and 8 m long. The 12 plots were arranged in three rows in the field, separated by 2 m of border space (Figure 3). The point cloud data collection system scanned the entire field, covering all 12 plots. The scan data for each individual plot were stored in a separate file on the industrial control unit.

### 2.4. Ground-Truth Data Collection

**Figure 4:**
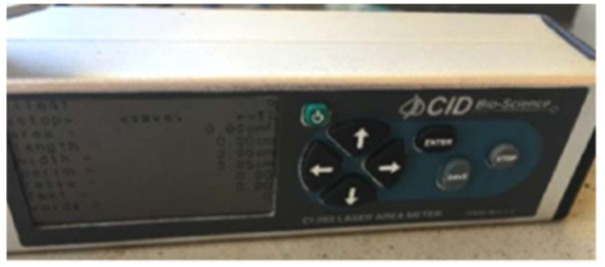
The leaf area meter was used to measure the leaf areas of rice plants.

To validate the estimated values obtained with the LiDAR, four 1 m × 1 m rice canopy parameter sampling areas were established within each of the 12 rice fields. These areas were designated for the measurement of the rice canopy height and the single leaf area of all rice plants in the sampling area. The sampling areas were uniformly distributed across each field plot. Rice plant heights were measured manually, while rice leaf areas were collected using a CI-203 Leaf Area Meter produced by CID, USA (Figure 4). This portable leaf area meter has a maximum measurement width of 102 mm, accuracy of <1% and a resolution of 0.05 mm^2^. The LAI for each field plot was calculated using Formula (1).

(1) $$\mathrm{LAI}=\frac{\sum_{i=1}^{4}\mathrm{LA}(n_{i})}{4}$$

In the formula, LAI represents the Leaf Area Index of the field plot, LA stands for the total leaf area of the plants within the sampling area and n_i_ denotes the identification number of the sampling area.

### 2.5. Raw Data

The raw data primarily consisted of distance, angle, timestamp and reflectivity information. The LiDAR was configured with their center as the origin, using horizontal rotation angles and distance parameters. The resolution of the horizontal rotation angle values was 0.01 degrees. The angle and distance information in the polar coordinates was transformed into Cartesian coordinates (xyz) in the LiDAR’s coordinate system. The defined timestamps were used to record the system’s time with a resolution of 1 ms.

The data also included reflectivity information about the measured objects. Reflectivity is the measure of an object’s ability to reflect light and is greatly influenced by the material properties of the object. During the collection of the rice canopy point cloud data, the LiDAR moved along the data collection system, continuously capturing the data at a sampling interval of 0.1 s.

### 2.6. Data Preprocessing

Each LiDAR dataset from the 12 plots was collected based on the predefined experimental setup and required preprocessing. The process thereof involved defining the region of interest and using the point cloud data collection system to scan with a horizontal width of 12 m while moving uniformly along the rows for 8 m within the plot. This scanning process captured the rice canopy point cloud data within each plot. The point cloud data were then converted from polar coordinates to Cartesian coordinates and integrated into a common reference coordinate system. Subsequently, the point cloud data were registered, overlapping points were trimmed and redundant points were removed.

**Figure 5:**
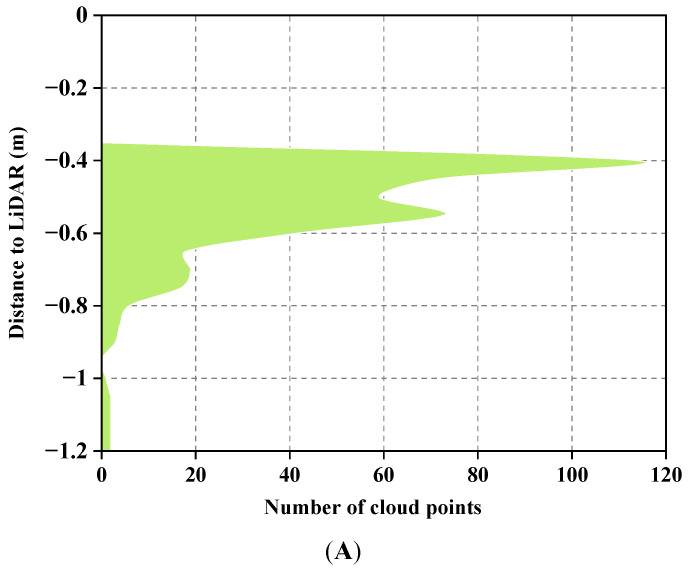

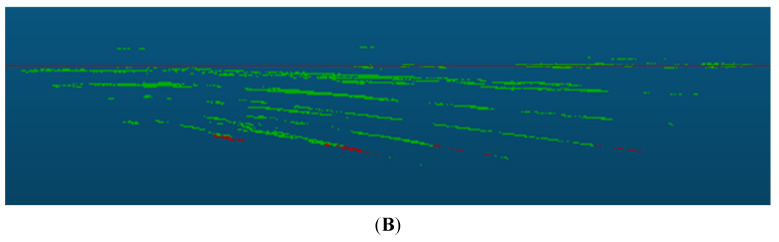
Illustrations of the determination of canopy height using LiDAR. (**A**) The distance–area accumulation plot relative to LiDAR. (**B**) A side view of the point clouds from the same column as (**A**). Non-ground points are represented by green point clouds, ground points are represented by red point clouds and the red line corresponds to the height at the percentile of 0.975.

Once integrated, the point cloud data of the region of interest appeared as shown in Figure 5, in which all the datasets have been combined and processed to depict the rice canopy information accurately.

#### 2.6.1. Rice Canopy Height Calculation

To determine rice canopy height using LiDAR, it is necessary to estimate the ground elevation and subtract it from the absolute height of the points. In this study, the nominal distance from the LiDAR to the ground was relatively fixed and the LiDAR’s position was above the rice canopy. The method used involved selecting the top point cloud of the canopy detected with the LiDAR using a percentile-based approach and calculating a surface model for the rice canopy [25]. The estimated canopy height was the difference between the ground elevation on the *Z*-axis and the percentile value.

To determine the optimal percentile value that would define the top of the rice canopy, a range of percentile values from 0.8 to 1, with increments of 0.005, was tested. Manual measurements of the canopy height were performed using a ruler [28]. The RMSE was calculated between the canopy height estimated with the LiDAR and that measured manually in each case. This provided an assessment of the accuracy of the LiDAR-based estimation of the canopy height compared to manual measurements.

#### 2.6.2. Rice Canopy LAI Estimation

The LAI estimation model was primarily determined through its correlation with the gap fraction derived from the LiDAR data [29]. The underlying theory thereof is based on the transformed equation of the Beer–Lambert law (Equation (2)).

(2) $$\mathrm{LAI}=-\frac{1}{k}\ln(\frac{I}{I_{0}})$$

In Equation (2), LAI stands for the Leaf Area Index, I represents the light intensity below the canopy, $I_{0}$ represents the light intensity above the canopy and k is the extinction coefficient.

Various laser penetration metrics (LPMs) were used as proxies for $I/I_{0}$ to estimate the LAI. For point cloud data, LPMs can be calculated as the ratio of ground echoes to total echoes or the ratio of ground echo intensity to total echo intensity [30,31].

To establish an LAI estimation model for rice canopies, a linear model following the Beer–Lambert law was used to compare LiDAR-derived LPM_1_ and LPM_2_. Regression analysis was performed between these LPM values and the LAI measurements obtained in the field. This regression analysis helped establish a relationship between the LPMs and the actual LAI of the rice canopy.

(3) $${\mathrm{LPM}}_{1}=\frac{N_{\mathrm{ground}}}{N_{\mathrm{total}}}$$

(4) $${\mathrm{LPM}}_{2}=\frac{S_{\mathrm{ground}}}{S_{\mathrm{total}}}$$

In Equations (3) and (4), LPM_1_ and LPM_2_ refer to LPMs. Specifically, LPM_1_ is the ratio of the number of ground echoes (N_ground_) to the total number of echoes (N_total_). LPM_2_ was calculated based on echo intensity and is the ratio of the intensity of ground echoes (S_ground_) to the total intensity of echoes (S_total_).

## 3. Results and Discussion

### 3.1. Rice Canopy Height Estimation and Accuracy Assessment

The LiDAR detection of the rice canopy height is illustrated in Figure 5. This analysis involved examining the *Z*-axis coordinates of all point clouds within the region of interest detected with the LiDAR. A histogram (Figure 5A) was constructed with the accumulated data of the maximum *Z*-axis values from the point clouds. Figure 5B provides a side view of the point clouds in the region of interest. In this representation, the blue line represents the ground obtained from the peak of the histogram, while the red line corresponds to the height at the percentile of 0.975.

**Figure 6:**
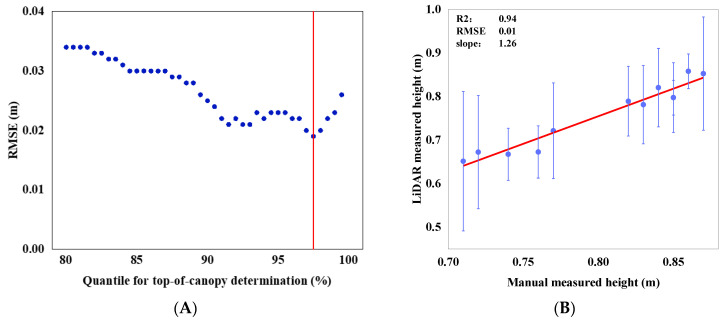
Validation of rice canopy height. (**A**) The RMSEs between manually measured canopy heights from different plots and those derived with LIDAR at different percentile values. (**B**) A scatter plot and a fitted plot comparing the average manually measured canopy heights from various plots with the canopy heights obtained with LiDAR detection at the percentile of 0.975. Error lines represent the positive and negative standard deviations for each plot.

From the point cloud side view in Figure 5B, it is evident that points closer to the ground are fewer in number. This is attributed to the dense canopy formed in the middle and later stages of rice growth, which reduces the likelihood of laser penetration into the canopy interior. Consequently, the majority of the point cloud concentrated on the upper part of the canopy. The results of the rice canopy height verification are shown in Figure 6. An analysis of the *Z*-axis coordinates of all point clouds within the region of interest detected with the LiDAR was conducted. Percentiles ranging from 80% to 100%were calculated with increments of 0.5%. The optimal percentile was determined by minimizing the RMSEs between the manually measured canopy heights and those obtained from various percentiles of the LiDAR detection. The optimal percentile was found to be 0.975 (Figure 6A). Linear regression analysis was performed between the manually measured and LiDAR-detected canopy heights. The coefficient of determination (R^2^) was 0.941, the RMSE was 0.019 m and the slope was 1.266 (Figure 6B).

A comparison between the manually measured average rice canopy heights from the different plots and those detected using LiDAR is provided here. Table 3 not only presents the detection errors between the two methods but also includes the standard deviations of the manually measured canopy heights. The minimum and maximum errors in the detected canopy heights were 0.24% and 12.98%, respectively.

This experiment has demonstrated that estimating rice canopy height through LiDAR is feasible, yet accurate ground elevation data are still crucial. Table 3 shows the differences between the manual and LiDAR measurements of rice canopy height, indicating that the height detected with the LiDAR was slightly lower than that measured manually. Due to the fact that rice is planted in wetlands, the LiDAR data acquisition platform remains stationary in the wetland, so it will cause the wheels to sink into the ground due to the pressure of its own weight. The ground elevation was determined based on the distance between the LiDAR and the ground at this time. While the platform is traveling, the degree of wheel sinking decreases, which increases the relative distance between the LiDAR and the ground. When calculating canopy height, the distance between the LiDAR and the ground used for calculation was a fixed value, and its absolute value may be smaller than the actual absolute value of the distance between the lidar and the ground. More lidar point clouds were ignored, and the distance between the ground and canopy point clouds decreased. This may be the reason for the underestimation of rice canopy height. Different plots had different bearing capacities for the LiDAR data collection platform. For example, in plot 9, the detection error for the height of the rice canopy was the smallest, at 0.24%, while in plot 4, the maximum detection error for the rice canopy height was 12.98%.

### 3.2. LAI Estimation Using LiDAR Data

The rice canopy point cloud data collected with the LiDAR were used to assess and compare two methods for estimating rice LAIs. The impacts of key parameters, namely two LPMs, on LiDAR-detected LAIs were evaluated. Regression analysis was conducted between the LiDAR-derived LAIs using the two LPMs and the field-measured LAIs. The coefficient of determination (R^2^) and the RMSE were calculated to evaluate the fitness of the constructed models. Table 4 provides the data for the two penetration metrics, as well as the manually measured LAIs and standard deviations.

**Figure 7:**
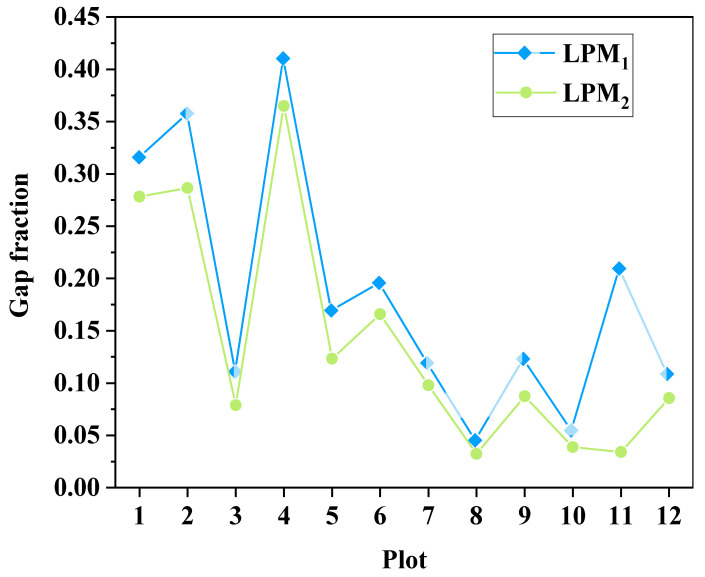
Comparison of gap fractions among different plots.

A factorial analysis of variance was conducted on the two different gap fractions for various plots to assess the correlation between those fractions. The results thereof indicated a strong positive correlation between the fractions (F = 12.866, *p* < 0.05). Figure 7 presents a comparative illustration of the gap fractions from different plots.

Different inter-row gap scores (LPM_1_ and LPM_2_) were subjected to factorial analysis of variance across different field plots to assess the correlation between these gap scores and the average rice LAI measured manually. The results indicated varying degrees of correlation between different gap scores and the average rice LAI. Specifically, there was a weak correlation between LPM1 and the rice canopy LAI (F = 4.599, *p* = 0.058 > 0.05), while LPM2 showed a stronger correlation with the manually detected rice canopy LAI (F = 3.499, *p* = 0.043 < 0.05).

A simple linear regression analysis was performed between the different plots’ gap fractions (LPM1 and LPM2) and manually measured average rice canopy LAIs. Different LPM analyses yielded varying R2 and RMSE values; for LPM1, R2 was 0.24 and the RMSE was 0.1, and for LPM2, R2 was 0.28 and the RMSE was 0.09. Previous LAI estimation models based on LPMs achieved variances of 69–94% for most studies, which were primarily focused on estimating forest canopy LAIs. Even for low-statured wetland vegetation (<2 m), variances of 55–60% in LAI estimation have been attainable [31]. In this study, the rice canopy height was less than 1 m. LiDAR’s capability to produce ground echoes in densely vegetated and low-statured canopy regions is limited due to its poor penetration ability in such environments [32]. In comparison to forests and relatively low-statured vegetation, rice is much shorter, and its dense canopy reduces the probability of laser pulses penetrating the canopy to reach the ground. This is a critical factor that affects the accuracy of LAI estimation using LPM.

**Figure 8:**
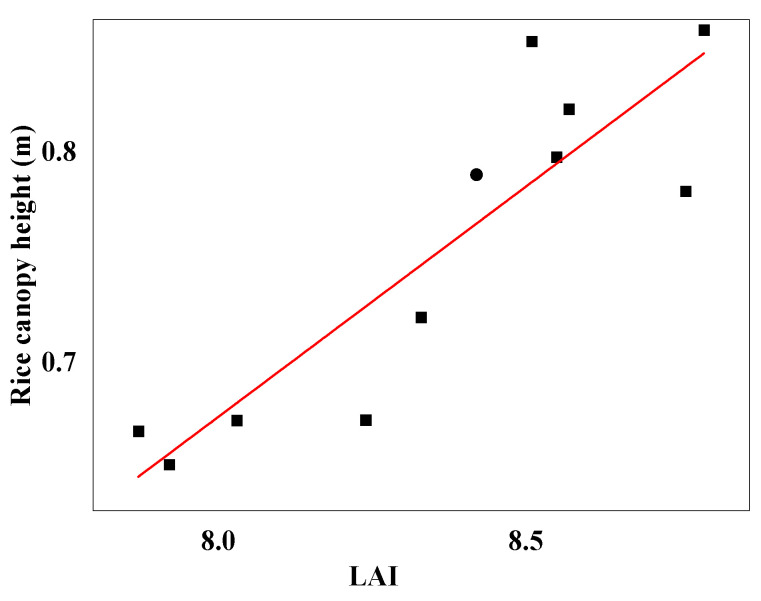
Relationship between rice canopy height and LAI.

In this study, a simple linear regression analysis was conducted between the manually measured LAIs from different field plots and the rice canopy heights detected with LiDAR. The obtained R^2^ value was 0.77, and the RMSE was 0.03. These results strongly indicate a significant correlation between rice canopy height and rice canopy LAI. A scatter plot of rice canopy LAI against LiDAR-detected rice canopy height is depicted in Figure 8.

## 4. Conclusions

This study demonstrated the feasibility of using LiDAR to detect rice canopy height and density. Through the analysis of LiDAR point clouds, accurate estimation of canopy height can be achieved. The use of gap fraction estimation for rice LAIs based on ground returns and return intensity showed a strong correlation between the different methods of gap fraction calculation. However, during the heading stage of the rice, the dense canopy reduced the likelihood of laser penetration to the ground, making it challenging to estimate the LAI using gap fractions, even though a correlation exists between LAIs and gap fractions based on return intensity. Moreover, as the density of the rice canopy further increased with the rice growth, the difficulty of detection became greater. The regression analysis of the rice canopy heights predicted with the LiDAR and the LAIs revealed the possibility of estimating LAIs based on the canopy heights of this variety of rice. In summary, LiDAR point clouds hold significant potential for the assessment of morphological parameters and distribution in crops, yet challenges persist in applications involving low-stature and dense crops like rice. This research has contributed to the improvement of the accuracy of estimating rice canopy heights and LAIs. Further studies are needed to enhance filtering algorithms for the accurate classification of low-stature and dense crops.

## Figures and Tables

**Table 1:** The LiDAR specifications.

Indicator	Value
Laser Beams	16
Range	20 cm~150 m
Range Resolution	+/−2 cm
Scan FOV	30° × 360°
Vertical Angle Resolution	2°
Rotation Rate	300/600/1200 (r/min)
Laser Wavelength	905 nm
Size	109 mm (diameter) × 82.7 mm (height)
Working Temperature	−10 °C~+ 60 °C
Weight	0.84 kg

**Table 2:** The spray boom sprayer specifications.

Indicator	Value
Size (mm)	3430 (length) × 1750 (width) × 2360 (height)
Track Width (mm)	1540
Minimum Ground Clearance (mm)	1055
Quality (kg)	880
Engine Power Rating (KW/rpm)	17.1/3600
Travel Speed (km/h)	0–11
Battery	12 V 45 Ah

**Table 3:** Detection and estimation results of average rice canopy height.

Plot	Average Rice Canopy Height (m)			
	Manually Measured	Std	Measured with LiDAR	Error (%)
1	0.72	0.13	0.67242	7.08
2	0.74	0.06	0.66734	10.89
3	0.83	0.09	0.78127	6.24
4	0.76	0.06	0.67266	12.98
5	0.85	0.04	0.79745	6.59
6	0.77	0.11	0.7214	6.74
7	0.84	0.09	0.8204	2.39
8	0.85	0.08	0.79758	6.57
9	0.86	0.04	0.85796	0.24
10	0.82	0.08	0.78918	3.91
11	0.87	0.13	0.85257	2.04
12	0.71	0.16	0.65143	8.99

**Table 4:** Measurement and estimation results of rice LAIs.

Plot	LAI of Rice (m^2^·m^−2^)			
	Measurement	Std	LPM1	LPM2
1	0.803	0.085	0.315385	0.278176
2	0.787	0.069	0.357357	0.286521
3	0.876	0.097	0.109817	0.078993
4	0.824	0.039	0.410256	0.364966
5	0.855	0.106	0.168514	0.123336
6	0.833	0.081	0.194831	0.165871
7	0.857	0.045	0.117978	0.0979711
8	0.855	0.093	0.0438413	0.0323046
9	0.879	0.104	0.121986	0.0874539
10	0.842	0.065	0.0533563	0.0388916
11	0.851	0.058	0.208683	0.0341212
12	0.792	0.046	0.107438	0.0856531

## Data Availability

The data presented in this study are available on request from the corresponding author. The data are not publicly available due to the data will still be used.
